# Poly(acrylamide/styrenesulfonic acid) hydrogels for effective removal of Cr(III) ions

**DOI:** 10.1038/s41598-025-30812-w

**Published:** 2025-12-18

**Authors:** Abdalla M. Khedr, Ashraf A. Lotfalla, Kazuhiro Hara, Satoru Yoshioka, Mohamed Gaber

**Affiliations:** 1 Chemistry Department, Faculty of Science, Tanta University, Tanta, 31527 Egypt; 2 Department of Applied Quantum Physics and Nuclear Engineering, Faculty of Engineering, Kyushu University, Fukuoka, 819-0395 Japan

**Keywords:** Hydrogel, Polyacrylamide, Styrene sulfonic acid, Chromium, Adsorption, Tanning, Chemistry, Environmental sciences, Materials science

## Abstract

Techniques for chromium ion removal have been researched more and more because of the significant harm to human health posed by this hazardous contamination in water. The present work represents the preparation of poly[acrylamide(Acr)/styrenesulfonicacid sodium salt (Sty)] hydrogels by free radical cross-linking polymerization applying various Acr and Sty monomer molar ratios. To produce Cr-poly(Acr/Sty) chelates, the produced hydrogels were examined for adsorption and desorption tests towards Cr(III) ions. The resulting hydrogels displayed a significant capacity for Cr(III) ion adsorption. Notably, the amount of Sty monomer in the hydrogel has a substantial effect on the Cr(III) ion capturing capacity. Cr-poly(Acr/Sty) and poly(Acr/Sty) hydrogels were characterized by modern analytical techniques such as X-ray diffraction (XRD), Energy Dispersive X-ray (EDX), scanning electron microscopy (SEM), Furrier transform infrared spectroscopy (FT-IR), and thermal gravimetric analysis (TGA). Research on the impacts of time and starting concentration of Cr(III) ions showed that hydrogels had the highest adsorption capacity for Cr(III) ions, at 346.48 mg g-1 dry gel. Hydrogels exhibited an exceptionally high swelling ratio, with a maximum value of 10490.46%. According to their adsorption kinetics, the findings are aligned with the pseudo second-order approach, and their adsorption performance coincides with the Freundlich isotherm. Their potential for reuse in environmental applications is demonstrated by the high desorption recovery percentage (almost 100 weight%), which could be used for the treatment of wastewater. Adsorption using Cr(III) from tanning industrial wastewater was conducted on the prepared hydrogels with efficient capturing capacity.

**Supplementary Information:**

The online version contains supplementary material available at 10.1038/s41598-025-30812-w.

## Introduction

One of the greatest environmental problems the world is currently facing is the lack of adequate wastewater treatment techniques^1,2^. Roughly 80% of all wastewaters is released back into the environment without being recycled or purified in any way^3^. Thus, a wide range of dangerous bacteria and infections taint the water, forcing approximately 1.8 billion people to consume it^4^. Instances of environmental and water contamination have been greatly worsened because of growth in human population, increasing energy output, and rapid promotional development^5^. These contaminants pose a significant risk for causing diseases and fatalities due to their poisonous nature, lack of biodegradability, and tendency to accumulate throughout the food chain^6^.

Numerous industrial activities, such as those in the plating and electroplating industries, mining, tanning, textiles, petrochemicals, and others, produce contaminated effluents. The management of the environment and public health is severely hampered by these activities, which can aid in the release of contaminants into the surroundings^7^. Due to their widespread usage in the chemical industry generally, industrial effluents are frequently characterized by the presence of many pollutants, both inorganic and organic^8^. These contaminants include heavy metals, nitrogen, phosphate, organic debris, hydrocarbons, chemicals that can upset endocrine systems, and pathogenic microbes^8–11^. Many heavy metals harmful to the ecosystem, such as (As), (Cd), (Cr), (Ni), (Cu), (Pb), (Hg), and (Zn), were recognized. As a resulting from anthropogenic sources, such as mining, metallurgical industry, mineral processing, leather tanning, electroplating, the dyeing industry, the oil industry, and other chemical industries, they are commonly attributed to the discharge of the hazardous heavy metals^12,13^. The toxicity levels exhibited by those heavy metals depend upon the dose and length of time that living organisms are exposed to them^13,14^. Heavy metals are hazardous and carcinogenic. These tend to collect within the cells of living organisms due to their non-biodegradable nature. This results in long-term bioaccumulation and environmental pollution, causing high risks to the ecosystems and human health^13,15^.

One of the most hazardous heavy metals that occurs naturally and is frequently utilized in industrial operations is Cr(III)^16^. It is a heavy metal that is non-biodegradable and has a propensity to build up in ecosystems, contaminating soil and water^17^. Prior to discharge, remediation procedures are required to raise effluents containing Cr ions to levels that comply with statutory regulations. The world Health Organization has set up a concentration limit of 0.1 mg L^- 1^ of chromium for water meant for human consumption^17^.

In fact, it is frequently observed that the concentrations of Cr ions in common wastewaters range from 50 to 100 mg L^- 1^, which is around 1000 times higher than the permissible limit. Therefore, before chromium is released into the environment, it is imperative that its levels be brought down to acceptable levels using the proper technology^18^.

Techniques including solvent extraction, ion exchange, coagulation, chemical precipitation, activated carbon adsorbent, and membrane processing, etc., may all be employed for pollution treatments. For effective and sustainable waste management, the processes offer practical ways to recover chromium from industrial tanning effluents^19^. Among those techniques, adsorption has the advantages of having a wide variety of target pollutants, being selective depending on adsorbent, having high capacity, being cost-effectiveness, having fast kinetics, and having the possibility of adsorbent regeneration and reuse, supporting sustainability. whereas the disadvantages Performance depends on type of adsorbent and chemical derivatization to improve its sorption capacity^14,20^. Adsorption methods need the selection step of an efficient adsorbent for certain pollutant removal, which is critical to decide its efficacy^20,21^.

Hydrogels are very attractive for cleaning up the environment. Their capacity to swell creates a vast network structure with a lot of surface contact, which facilitates the diffusion of pollutant molecules and improves adsorption^22^. Additionally, their physical and chemical characteristics can be carefully manipulated during synthesis to include functional groups that have a strong affinity for particular kinds of pollutants, making it possible to create effective and selective adsorbents^14^.

Particular functional groups on the surfaces’ adsorbent are thought to improve the adsorption, such as carbonyl, -SO_3_Na, and amide groups. The amide groups develop a covalent crosslinking network within the material, thereby giving an effective adsorption process, where the number of functional groups in the hydrogels’ structure increases several times their potential to absorb metals^23,24^.

The three-dimensional network structure of the hydrogels makes them able to absorb and hold significant volumes of water inside their structure without dissolving. They are distinct from other material groups due to their high-water content, high cross-linking, hydrophilicity, and high swelling capability^25–27^. Because of their unique design, these hydrogels exhibit significant volumetric swelling in response to a variety of environmental stimuli, such as variations in pH, temperature, and ionic concentration. As a result, they are very adaptable for a wide range of applications^26,28^.

To purify industrial wastewater that contains heavy metals like Cr(III), which is utilized in the petrochemical sector, certain hydrogels of polymers containing an ionic component can collect hazardous metallic ions^25,29^. By attaching an operational ionized group to the core of the polymer matrix, the hydrogels exhibit intriguing features. The hydrogels’ exceptional effectiveness in adsorbing heavy metals and their significant benefit as an environmental purifying material have been noted by Hara et al.^30^. So, researchers are interested in examining the effectiveness of different hydrogels in collecting toxic metals in previous investigations. They discovered that, in addition to the hydrogel’s reusability^26^, nearly all their activities in collecting heavy metals are significantly higher than those of ion-exchange and zeolite resins^25,31^. Trivalent chromium and other metallic elements are found in wastewater from industries, for instance, the tanning industry, which releases large amounts of wastewater containing chromium with other contaminants that definitely will pollute the environment and harm health and disturb the ecosystem. Trivalent chromium in the wastewater could be easily oxidized to the more highly toxic hexavalent chromium^2,30^. Exposure to Cr(VI) may cause cancer, respiratory issues, skin rashes, hemolysis, acute renal failure, compromised immune systems, liver and kidney damage, lung cancer, pulmonary fibrosis, gastrointestinal ulcers, and dermatitis^2,30–32^. This work affixes an essential ionized group to polymeric hydrogels’ core, which have an extremely large swelling proportion and are porous structures. To reduce the health and environmental hazards that could arise from Cr(III) seeping into the aquatic environment and other environmental components, such concerns highlight the need for chromium extraction and reuse. As a result, human health is preserved, and the environment remains sustainable. Applying this technique, we will be able to establish highly efficient Cr(III) removal capacity, and the number of possible reuses will shift to a low-cost adsorbent poly(Acr/Sty) hydrogel. With the optimal molar ratio, that will reduce the cost of Cr(III) removal and prevent the penetration of chromium metal ions into the environment. making it a highly sustainable and economically feasible adsorption process.

## Experimental

Acrylamide (Acr) (99%), styrenesulfonic acid sodium salt (Sty) (98%), NMBA (N, N’-methylene-bis-acrylamide) (99%) crosslinker, and AP (NH_4_)_2_S_2_O_8_) (98%) initiator have been purchased from Sigma-Aldrich. Hebei Chromate Chemical Co., Ltd. supplied basic chromium sulfate (Cr(OH)SO_4_) (98%).

### Poly (acrylamide/styrene sulfonic acid) hydrogel (Acr/Sty) preparation

Poly(acrylamide/styrene sulfonic acid sodium salt) hydrogels (poly(Acr/Sty)) have been produced applying free radical polymerization. The deionized water (DI) was used to synthesize the pre-gel solution with total polymer concentrations of 0.7 M, 1.4 M, 2.1 M, and 2.8 M and molar ratios of each monomer in the 6:1–4:3 [Acr: Sty] range. As a crosslinker, 0.133 weight% of NMBA (N, N-methylenebisacrylamide) is incorporated into every pre-gel solution. The polymerization (gelation) reaction was initiated by employing a polymerization initiator of 0.04 weight% (NH_4_)_2_S_2_O_8_^30^.

Regarding the first step in the production process, it was to dissolve Acr in 50.0 milliliters (DI) at room temperature. For full dissolution and continue stirring for up to 20 min, it was agitated utilizing a magnetic stirrer. Sty was added to the solution under the same circumstances. NMBA was added when sufficient dissolution was ensured, with about 20 min spent stirring the mixture. The initiator, (NH_4_)_2_S_2_O_8_, was then added and swirled until completely dissolved. Stirring continued for twenty more minutes. To synthesize gels, after carefully pouring the pre-gel solution into a Coplin jar, it was capped, sealed, and incubated for 24 h at 60 °C.

**Fig. 1:**
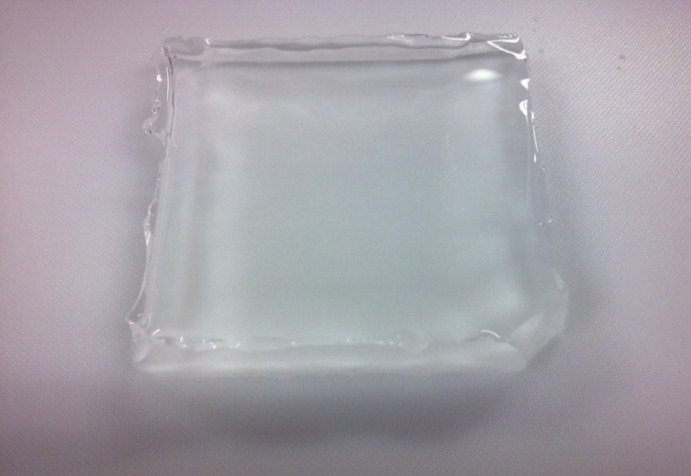
The shape of the prepared poly(Acr/Sty) hydrogel.

Following gelation, with caution, the pre-gel was removed from the Coplin jar and rinsed three repeated times using DI to get rid of any unreacted materials. To induce swelling, it was then submerged in DI for a whole day. The produced poly(Acr/Sty) hydrogel with varied monomers molar dosing concentration was labeled from G1 to G12. Figure 1 shows the shape of the synthesized poly(Acr/Sty) hydrogel.

## Adsorption of chromium ions

An inventory of 500 ppm of Cr(III) was produced to study the poly(Acr/Sty) gel’s capturing tendency towards Cr(III) ions employing Cr(OH)SO_4_ (basic chromium sulfate), which is employed in the leather tanning industry. The section of the gels that had swollen was cut into a cylindrical shape with around a 30 mm diameter and a height range of 0.6 mm to 1.4 mm. It was then weighed, immersed in fifty milligrams of 500 ppm Cr(III) in a vessel, and left for time intervals of 10 min, 20 min, 30 min, and 1, 2, 3, 4, 5, to24 hours. Cr(III)-adsorbed hydrogels were created by carefully removing each gel from the solution after each cycle. In accordance with each of the previously manufactured hydrogels (G1 to G12), The hydrogels of Cr-poly(Acr/Sty) that were produced were given the designations CG1 through CG12. The amount of Cr(III) in the leftover solution was decided using the atomic absorption spectrometer (AAS)^33^. Equation (1) was used to determine the quantity of Cr(III) absorbed by hydrogel^25,30^. Using varying molar ratios of the produced hydrogel, the impact of (Sty) monomer concentration on the ability of poly(Acr/Sty) hydrogel to capture Cr(III) was computed.

1 $$\:{{q}}_{{e}}=\frac{{({C}}_{{i}}-{{C}}_{{e}}){V}}{{m}}$$

where q_e_ in mgg^− 1^ stands for the quantity of Cr(III) that is absorbed, C_i_ in mg/L represents the starting concentration of Cr(III) ions, C_e_ in mg/L represents the level of Cr(III) ions at equilibrium, V in Liters represents the solution volume, whereas m in grams represents the hydrogel mass.

## Solid samples preparations

All produced hydrogels (poly(Acr/Sty)), G1-G12 and CG1-CG12 (Cr-poly(Acr/Sty)) hydrogels solid samples were subjected to drying and dewatering in a drying oven at 40–60 °C for 72–96 h. After that, a mortar was utilized to grind the samples into a powder that was prepared for the study’s research.

## Fourier transform infrared (FTIR)

The generated Cr-poly(Acr/Sty) chelates and poly(Acr/Sty) hydrogels were characterized by FTIR analysis^34^. FTIR spectroscopy is one of the most widely used analytical methods and has been used in many studies involving cross-linked polymeric materials^25,35^. In this study, FTIR spectroscopy was employed to verify that crosslinkers and monomers were incorporated into the hydrogel structure of polymers and to look into the interactions between polymer functional groups and Cr(III) ions in the interconnected structure of the hydrogel^36–38^. Using a KBr disc in the 4000–200 cm^− 1^ wavenumber range, a Bruker Transor 27 FT-IR spectrophotometer, structural analyses were performed.

## Morphological structure

A scanning electron microscope, type A JEOL JSM-5200 LV (Japan), was applied for SEM imaging, allowing for high-resolution investigations of the hydrogels’ microstructural characteristics. An SPI-ModuleTM Vac/Sputter was used to coat the samples in gold after they were fixed to a stub of metal. SEM was applied to examine the hydrogels’ morphology, surface components, size, porosity, and cross-linking^26,29^. This technique made it possible to identify structural differences and conduct a detailed comparison of the surface morphologies of Cr-poly(Acr/Sty) chelates and poly(Acr/Sty) hydrogel^25,36,39^.

### (TGA)studies

The thermal analyzer Shimadzu TGA-50 Series (Japan) was used to characterize the maximum capacity to capture Cr(III) of GC3 (Cr-poly(Acr/Sty)) and G3 hydrogel in atmospheric nitrogen, with ten degrees Celsius per minute of heating, and the heating spectrum within the ambient temperature to 850 °C range^39^.

## Crystalline nature

With an 8°/min scanning speed and a Cu-pulse lamp operating at 40 kV with 30 mA, the XRD-6000 SHIMADZU X-ray diffractometer was applied to perform x-ray diffraction patterns in an angle range of 5° to 80°. The crystalline nature and phase can be characterized using XRD techniques, which are useful, adaptable, and non-destructive analytical methods. This technique made it possible to compare GC3 (Cr-poly(Acr/Sty)) with its G3 hydrogel and identify structural differences^38,40^.

## Hydrogel swelling ratio

The swelling behavior of hydrogels was investigated gravimetrically by applying the swelling experiment. After being weighed, At room temperature, the dried gel was immersed in deionized water for a whole day until it reached equilibrium. After taking the swelled hydrogel out of the water, it was carefully dried with filter paper to get rid of any remaining water before being weighed. Next, using the subsequent Eq. (2)^37,41^:

2 $$\:{S}{w}{e}{l}{l}{i}{n}{g}\:{r}{a}{t}{i}{o}\:\left({\%}\right)=\:\:\frac{{{W}}_{{s}}-{{W}}_{{d}}}{{{W}}_{{d}}}\:\times 100$$

where W_d_ is the dry hydrogel mass (g) and W_s_ is the swelling hydrogel mass (g) at equilibrium.

### Kinetics of adsorption processes

The rate for adsorption of the solute molecules on the adsorbent was ascertained by analyzing the adsorption of the non-linear forms by pseudo second-order and pseudo first-order kinetics approaches. Experiments on adsorption were carried out in relation to reaction time until equilibrium. should use Eqs. (3) and (4)^34,42^ to determine each model’s appropriateness.

3 $$\:{{q}}_{{t}}={{q}}_{{e}}(1-{{e}}^{-{{K}}_{1}{t}})$$

4 $$\:{{q}}_{{t}}=\frac{{{K}}_{2}{{q}}_{{e}}^{2}\text{t}}{1+{{{q}}_{{e}}{K}}_{2}{t}}$$

where k_1_ (min^− 1^) and k_2_ (g/mg min) represent the first- and pseudo second-order rate constants, respectively, and q_e_ and q_t_ represent the amounts of Cr(III) ion (mg g^− 1^) adsorbed at equilibrium and at time t in minutes, respectively.

### Influence of initial cr(III) ion concentration

Since the concentration of the sorbate is among the most crucial factors that might influence its adsorption on the sorbent, the impact of the beginning Cr(III) concentration was examined. Investigations using hydrogel G3 were carried out for a broad range of Cr(III) ion concentrations, from 10 to 500 mgL^− 1^. This was done at ambient temperature, and after a day, there was adequate time for equilibrium. The total quantity of Cr(III) adsorbed by the hydrogel was computed assuming Eq. (1)^26,42^.

### Adsorption isotherms

The most well-known models for assessing sorbents are the Freundlich and Langmuir isotherm models. These approaches were utilized extensively to determine the affinity of the surface and characteristics of adsorbents, which provide a rational understanding of the abilities to bind a certain adsorbate in a solution of water. They allow the computation of the adsorbent’s maximum theoretical capacity toward the target adsorbate and describe the characteristics of interfacial adsorption^25,26,40,42^.

The Langmuir model, which describes chemical adsorption, is based on monolayer adsorption on a homogeneous surface with equal sorption energy binding sites. When there is no interaction between the adsorbate molecules on the adsorbent surface, this model theoretically depicts the equilibrium distribution of the sorbate between the solid and liquid phases. A concentration variation serves as an adsorption-promoting element, and the adsorbent’s accessible surface area determines the adsorption intensity^25,40^. This model’s non-linear version was used in accordance with Eq. (5).

5 $$\:{{q}}_{{e}}=\frac{{{q}}_{{m}{a}{x}}{{C}}_{{e}}{{K}}_{{L}}}{1+{{C}}_{{e}}{{K}}_{{L}}}$$

where q_e_ is the amount of sorbate adsorbed per gram of adsorbent at equilibrium (mg g^− 1^), q_max_ is the adsorbent’s maximum adsorption capacity (mg g^− 1^), C_e_ is the equilibrium adsorbate concentration (mg L^− 1^), and K_L_ is the Langmuir isotherm that is continuously linked to the affinity (energy) of the binding site (L mg^− 1^).

The separation factor (R_L_), a dimensionless equilibrium constant that characterizes the properties of the Langmuir adsorption isotherm, is its main feature. R_L_ was computed to assess the potential adsorption of the Cr(III) metal ion on the G3 hydrogel surface. (R_L_) could be represented by Eq. (6)^26,43^:

6 $$\:{{R}}_{{L}}=\frac{1}{1+{{K}}_{{L}}{{C}}_{{e}}}$$

The value of (R_L_) is the Langmuir adsorption isotherm’s primary feature that defines the type of isotherm. Depending on whether R_L_ is greater than 1, equal to 1, or equal to 0, the adsorption process can be irreversible, linear, or unfavorable. Conversely, the adsorption process is seen advantageous if R_L_ values are within 0 < R_L_<1 range.

According to the Freundlich isotherm model, the sorbent must have a multilayer adsorption system, energy and reversible adsorption, and a nonuniformed (heterogeneous) surface^25,42^ Eq. (7) describes the Freundlich isotherm in non-linear form^40,42^:

7 $$\:{{q}}_{{e}}={{K}}_{{f}}{{C}}_{{e}}^{1/{n}}$$

where the Freundlich constant, K_f_ (mg/g), is used, representing the sorbent’s ability for adsorption. The dimensionless parameter n is associated with the intensity of adsorption. The heterogeneity factor, term 1/n, provides information about how well the adsorption process works when the sorbent is in equilibrium and binding. The adsorption procedure is advantageous under the specified operating conditions if 0 < 1/*n* < 1^42^.

### Desorption of Cr(III) experiment

To guarantee the release of Cr(III) ions in desorption of Cr(III) studies, 50 milliliters of 3 N HCl were treated with hydrogel for a full day. After that, 2 N NaOH was used to renew the hydrogel. The quantity of Cr(III) released from the hydrogel was counted using an atomic absorption spectrometer, and the tested concentration was multiplied by the solution’s volume to estimate the total amount of Cr(III)^44^. Remarkably, even after the regeneration process, this hydrogel managed to regain its cylindrical shape.

## Results and discussion

### Chromium ion adsorption study

It was discovered that the length of time of immersed hydrogel in the chromium solution affects the amount of Cr(III) adsorbed by the poly(Acr/Sty) hydrogel’s binding sites. After 24 h, there was a noticeable rise in the Cr(III) concentration that was adsorbing, reaching equilibrium. How much Cr(III) was adsorbed for each produced poly(Acr/Sty) hydrogel at each time interval is displayed in Table S1.

**Fig. 2:**
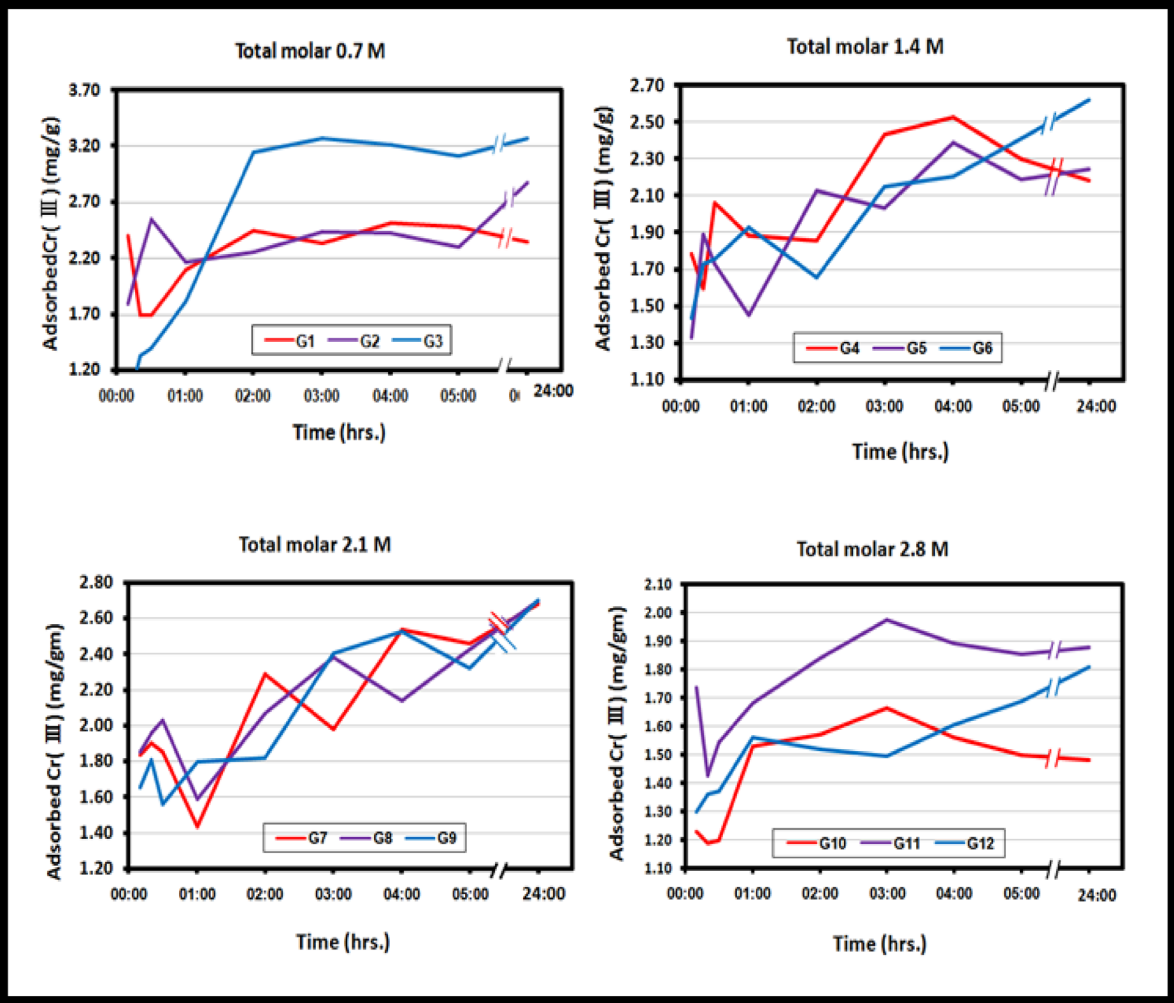
The distinctive Cr(III) adsorption curves for each produced poly(Acr/Sty) hydrogel of total molar concentrations 2.8 M, 2.1 M, 1.4 M, and 0.7 M versus time (10, 20, and 30 min and 1, 2, 3, 4, 5, 24 h).

**Fig. 3:**
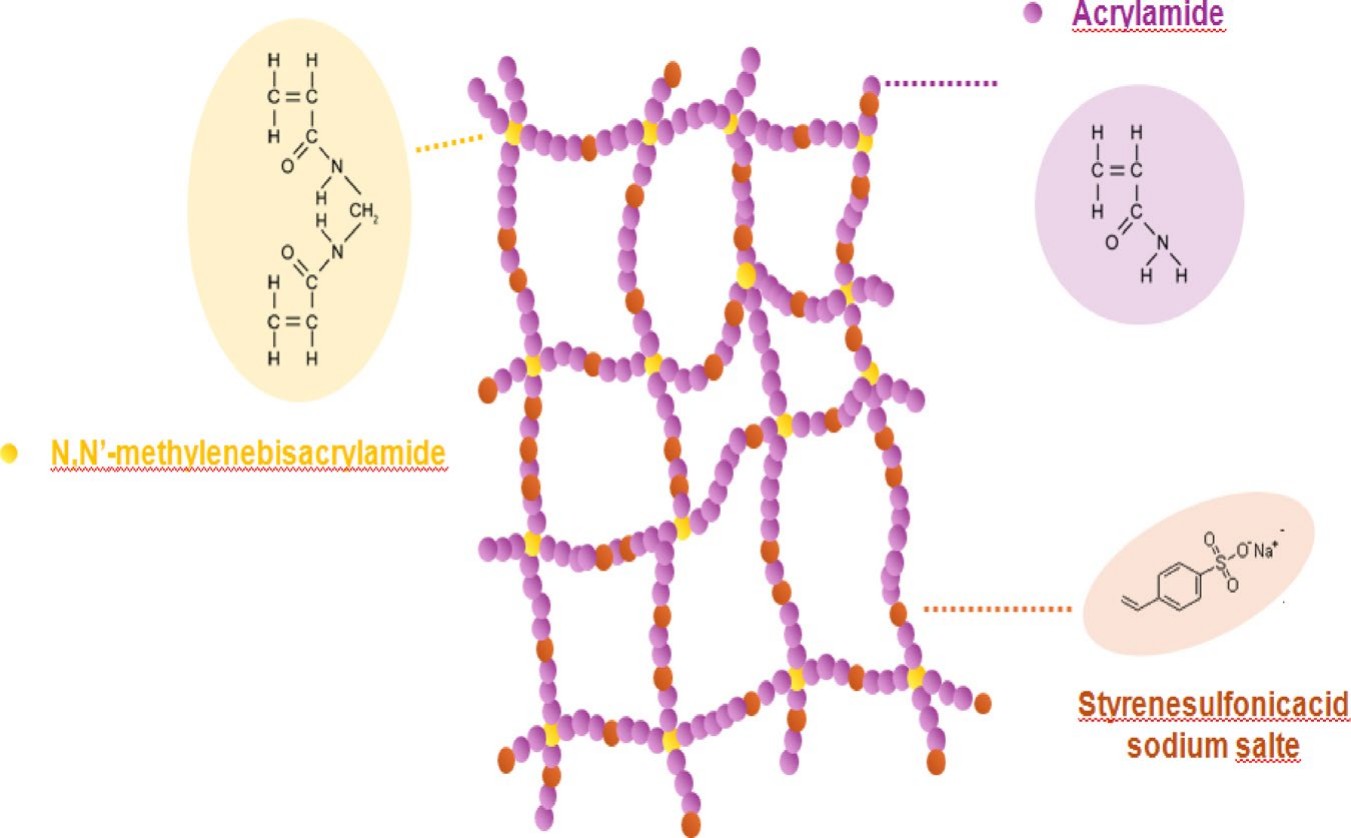
Configurations of the poly(Acr/Sty) hydrogel’s monomers with potential network structure.

Figure 2 shows the characteristic adsorption curves for G1-G12 (all manufactured poly(Acr/Sty) hydrogel) with molar concentrations overall of 2.8 M, 2.1 M, 1.4 M, and 0.7 M in relation to time (10, 20, and 30 min and 1, 2, 3, 4, 5, 24 h). In general, Cr(III) adsorption rises with time until it reaches its maximal capacity 24 h later. The poly(Acr/Sty) gel tends to adsorb Cr(III), and the concentration of Cr(III) causes the swelling water to be released from the hydrogel. During the adsorption process, the swelling water of the hydrogel is released, causing dilution of the remaining Cr(III) solution and giving rise to the distinctive adsorbing curves. The adsorption capacity depends on the hydrogel’s composition and the number of the binding sites (functional group (-SO_3_^−^) and other binding groups (-NH_2_ and -C = O). Figure 3 displays the possible structure of the networks of the hydrogel and the specific chemical compositions of the employed monomers.

### Impact of Sty-monomer concentration on Cr(III) adsorption

**Fig. 4:**
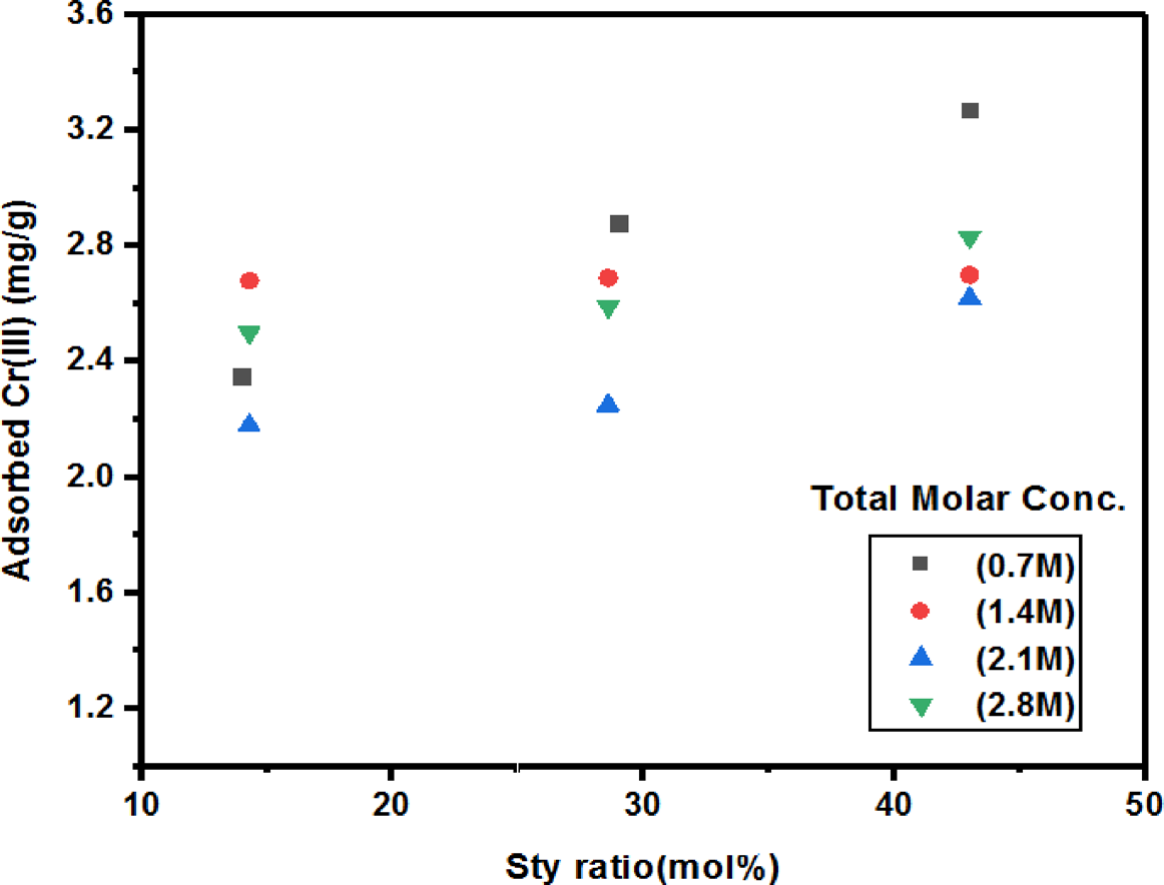
The Sty-fraction dependence of poly(Acr/Sty) hydrogels of total molar concentrations 2.8 M, 2.1 M, 1.4 M, and 0.7 M on Cr(III) adsorption.

How the hydrogels’ monomer (Sty) compositions affected their capacity to absorb Cr(III) was tested. The findings showed that the composition of the poly(Acr/Sty) hydrogels clearly affected the Cr(III) adsorption. Figure 4 illustrates the plotting of the Sty-percentage (mol%) against the quantity of Cr(III) that was caught (mg per gram of hydrogel)^2,30,45^. To a certain degree, the amount of captured Cr(III) rises as the Sty-fraction grows. After that, the amount of captured Cr(III) starts to fall, exhibiting a (mound-like) reliance on the concentration of the network. Results indicated a clear dependence of the Cr(III) adsorption on the poly(Acr/Sty) hydrogel’s composition^2,19,31^.

**Fig. 5:**
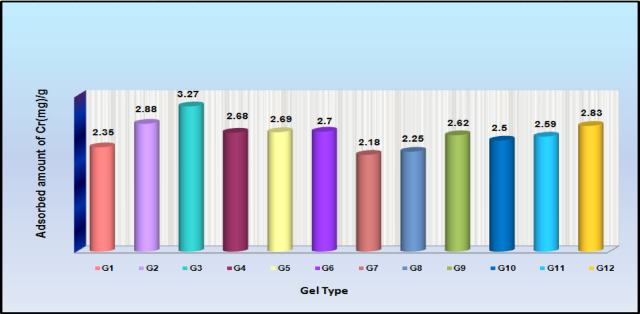
Evaluating the quantity of Cr(III) absorbed by each prepared hydrogel till equilibrium(24-hour period).

**Fig. 6:**
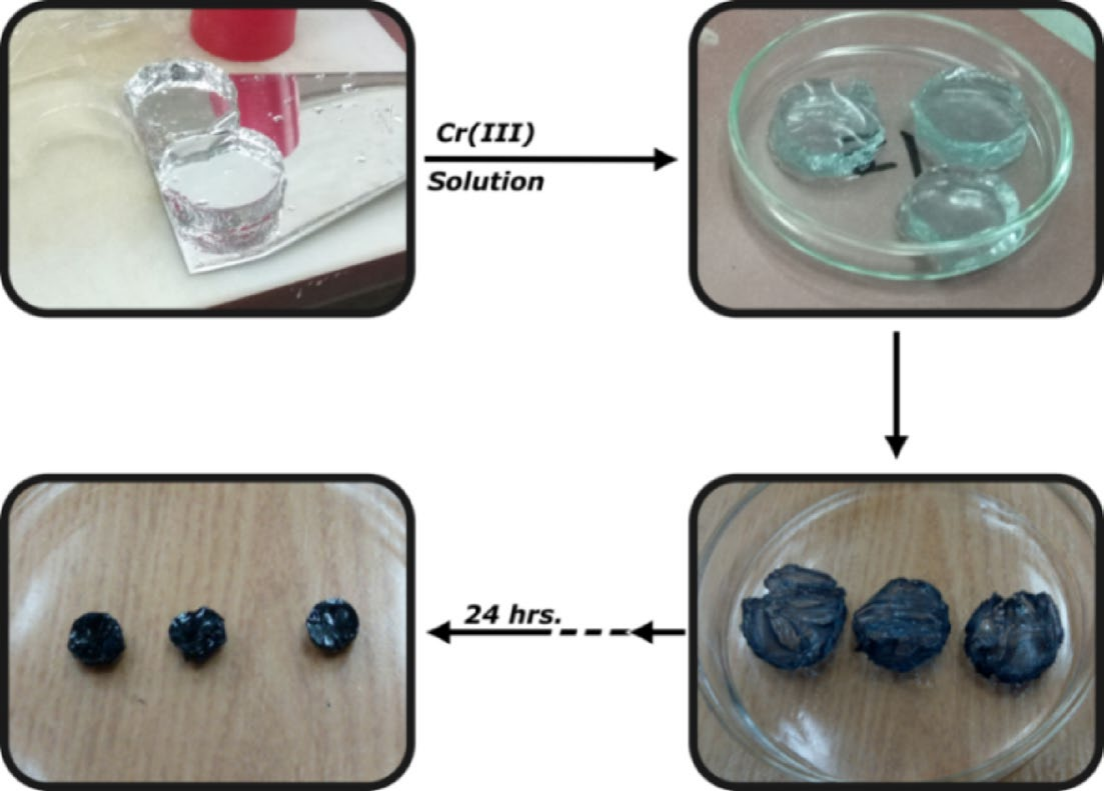
The morphological shape of poly(Acr/Sty) hydrogels after each immersion time till-24 h.

Figure 5 shows a graphical comparison of the quantity of Cr(III) collected by each of the generated poly(Acr/Sty) hydrogels. For Acr: Sty monomers with a molar ratio of 1.5:0.6 M, the maximum quantity was found at 2.1 M total molar concentration for the Cr(III) capture. It was discovered to be 3.27 mg/g of hydrogel that had swelled. Since the differences in their adsorption capacities may be attributed to the differences in their monomer’s molar constituents and the Sty-fraction, this indicates that the total molar ratio of 0.7 M with monomer constituents (0.4:0.3) allows better bounding sites than the others. Over time, there was a noticeable shift in the hydrogel’s (cylindrical samples’) shape. As seen in Fig. 6, they start to shrink after each time interval and darken in color as the amount of time they are submerged in the chromium solution increases. After 24 h, they achieve the gel’s maximal adsorbing capacity^2^.

### FT-IR structural investigation

To ascertain the structures of the produced hydrogels and the monomers, the FTIR utilized to examine the useful groups found in their spectrum. Figure 1S displays the spectra of the G1–G12 (poly(Acr/Sty) hydrogels) and the monomers (Acr, Sty, and NMBA) that were utilized. Table S2 documents the assignment of the bands that are being examined. For Acr, Sty, and NMBA attributed to symmetric and asymmetric and stretching, the IR spectra revealed peaks in the region (3107 − 3048) cm^− 1^ in line with the C = C group^46^. However, these peaks vanished in the spectrum of all the hydrogels, signifying the creation of polymeric hydrogels. In the polymeric chain of the hydrogels, the distinctive absorption peaks of the (CH_2_, CH) groups emerged at 2925–2937 and 2854–2866 cm^− 1^ ranges attributed to stretching and bending vibration of methylene^33,47^. Regarding the Acr and NMBA spectra, the NH_2_ and NH groups are represented by the peaks that appeared at 3351 and 3308 cm^− 1^, respectively; the NH_2_ peaks are still present in the hydrogel’s spectrum, but they change to a wavenumber that is longer at the 3429–3448 cm^− 1^ range, which is attributed to asymmetric stretching vibration^48,49^. The 1675 and 1660 cm^− 1^ peaks in the Acr and NMBA spectra, respectively, show the C=O groups linked to stretching vibration^50–52^. For the hydrogels, they also showed up, but they moved to a lower wavenumber in the 1668–1670 cm^− 1^ region^53^. The C-SO_3_ spectrum’s asymmetric stretching vibration was attributed to peaks in the Sty spectra that showed up around 690 cm^− 1^ wavenumber^54^. These peaks shifted to a wavenumber lower in the hydrogel’s spectra designated for stretching vibration, which were observed in the range 682–688 cm^− 1^. For the transmittance peaks of Sty assigned for stretching vibration, the peaks appeared at 1132 and 1188 cm^− 1^ attributed to S=O^55,56^. These peaks are still present in the hydrogels’ spectra, but their wavenumber shifts to the ranges of 1190–1199 and 1118–1128 cm^− 1^. They showed up, but they were moved to the hydrogels’ spectra’s lower wavenumber at range 1602–1612 cm^− 1^, this is the distinctive C_Ar_–C_Ar_ absorption band at 1630 cm^− 1^ of Sty^55^. The hydrogels’ spectra showed peaks at 1400 cm^− 1^, which corresponded to stretching vibrations in the aromatic skeleton of styrene^55^. However, the hydrogels’ synthesis was indicated by a shift to a longer wavenumber in the range of 1409–1413 cm^− 1^.

### Morphological inspection (SEM)

Because hydrogels are known to be porous, their cross-linked network and swelling properties cause them to fill with water. This raised the possibility that their surface and network structure had pores, cavities, etc.

**Fig. 7:**
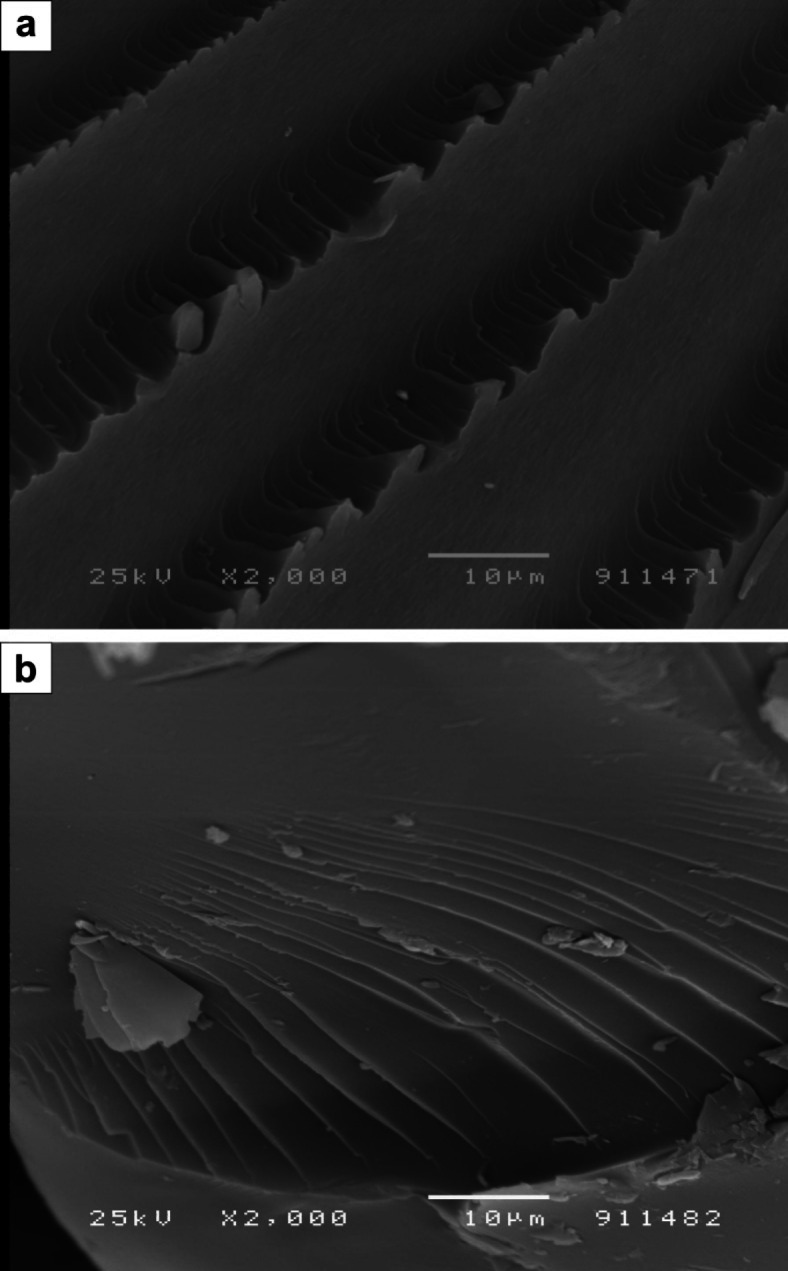
SEM images for (**a**) p(Acr/Sty) hydrogel and (**b**) p(Acr/Sty) hydrogel adsorbing Cr(III).

Figure 7 (a) displays the SEM pictures of the poly(Acr/Sty) hydrogel and the Cr-poly(Acr/Sty) chelate. They reported that, in contrast to the (a) poly(Acr/Sty) hydrogel, the surface of the (b) Cr-poly(Acr/Sty) chelate was observed to be more flatter in shape. The creation of the Cr-poly(Acr/Sty) could be the cause of this^56,57^.

### EDX analysis

**Fig. 8:**
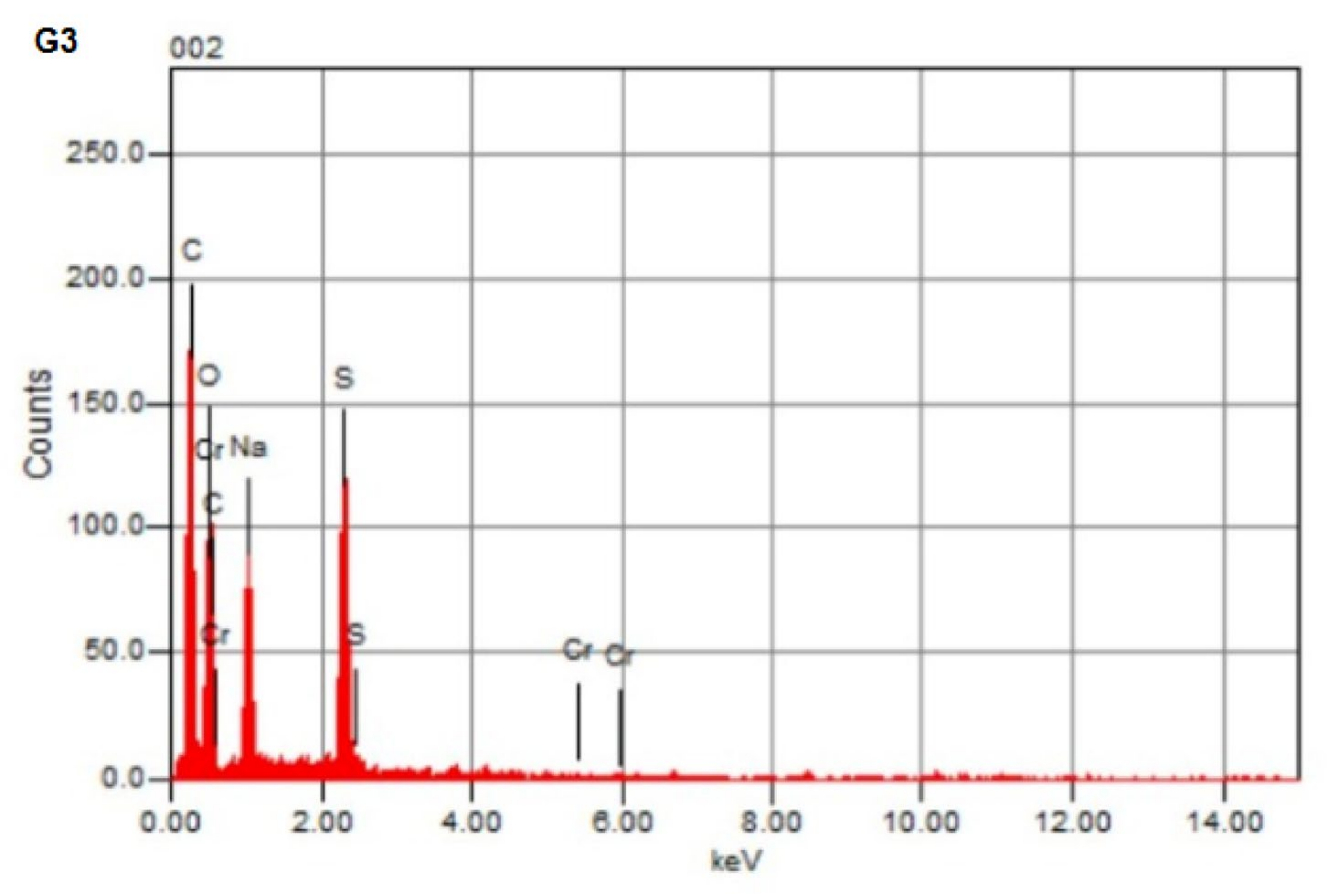

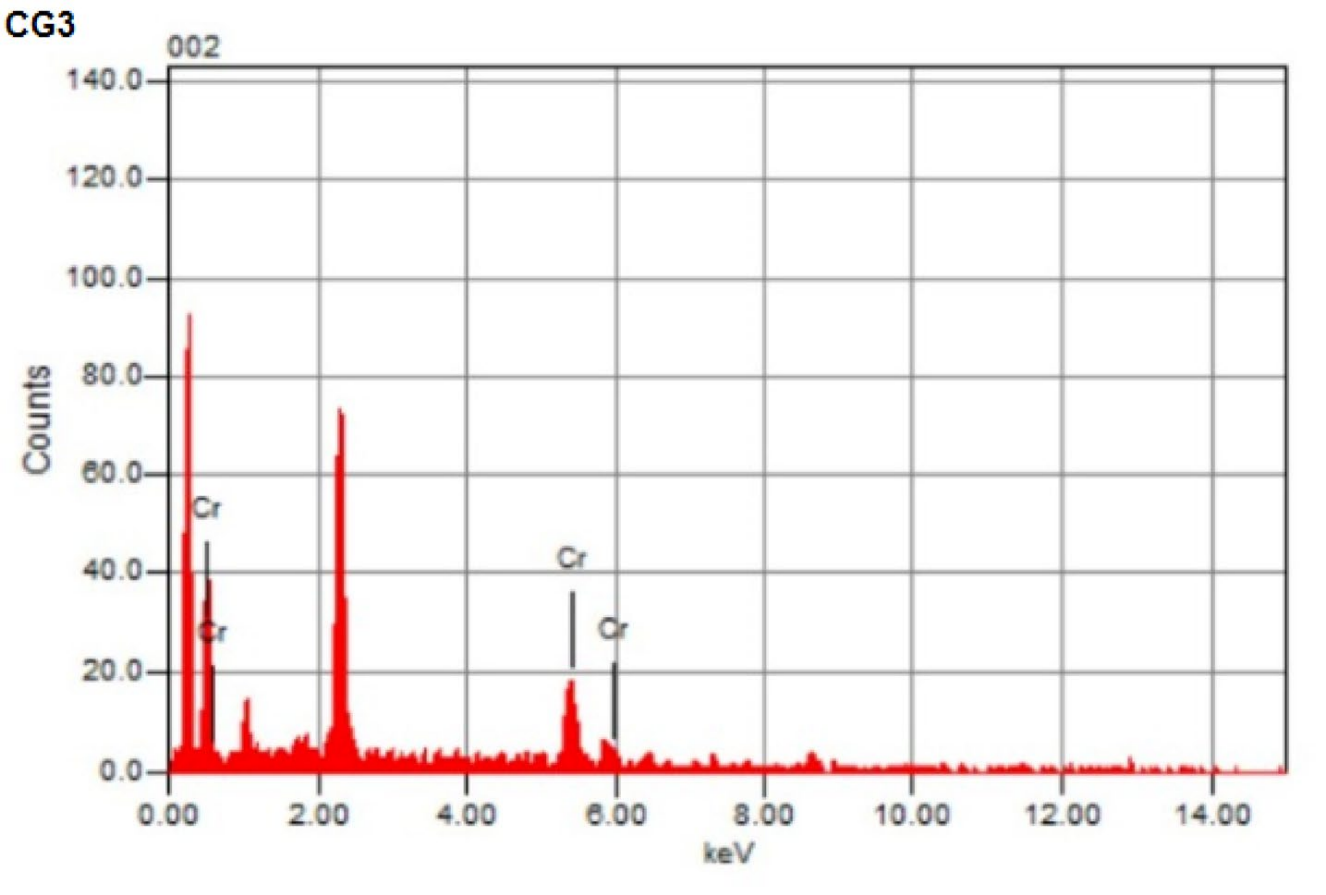
EDX of p(Acr/Sty) hydrogel (G3) before and after Cr(III) adsorption (CG3).

Along with the results, the adsorption of heavy metal ions Cr(III) onto poly(Acr/Sty) hydrogel was further confirmed by EDX analysis shown in Fig. 8, which displays the distinct signals indicating the adsorption of Cr(III). Also, EDX results showed Cr(III) content in hydrogel percentages, which is basically attributed to the adsorption onto the hydrogel matrix, confirming the successful adsorption of the Cr(III) metal ion onto the hydrogel’s surface, forming Cr-poly(Acr/Sty)^15,58^.

### TGA analysis

The examination and the determination of some important parameters pertaining to the hydrogel’s thermal stability were conducted using thermal gravimetric analysis (TGA). These include T_h_ (temperature of reaction half-life), T_f_ (temperature of reaction end), T_i_ (temperature at which thermal deterioration begins), T_m_ (maximum temperature of deterioration), rm (maximum degradation rate), and c_m_ (quantity of material remaining at the highest rate)^59^.

**Fig. 9:**
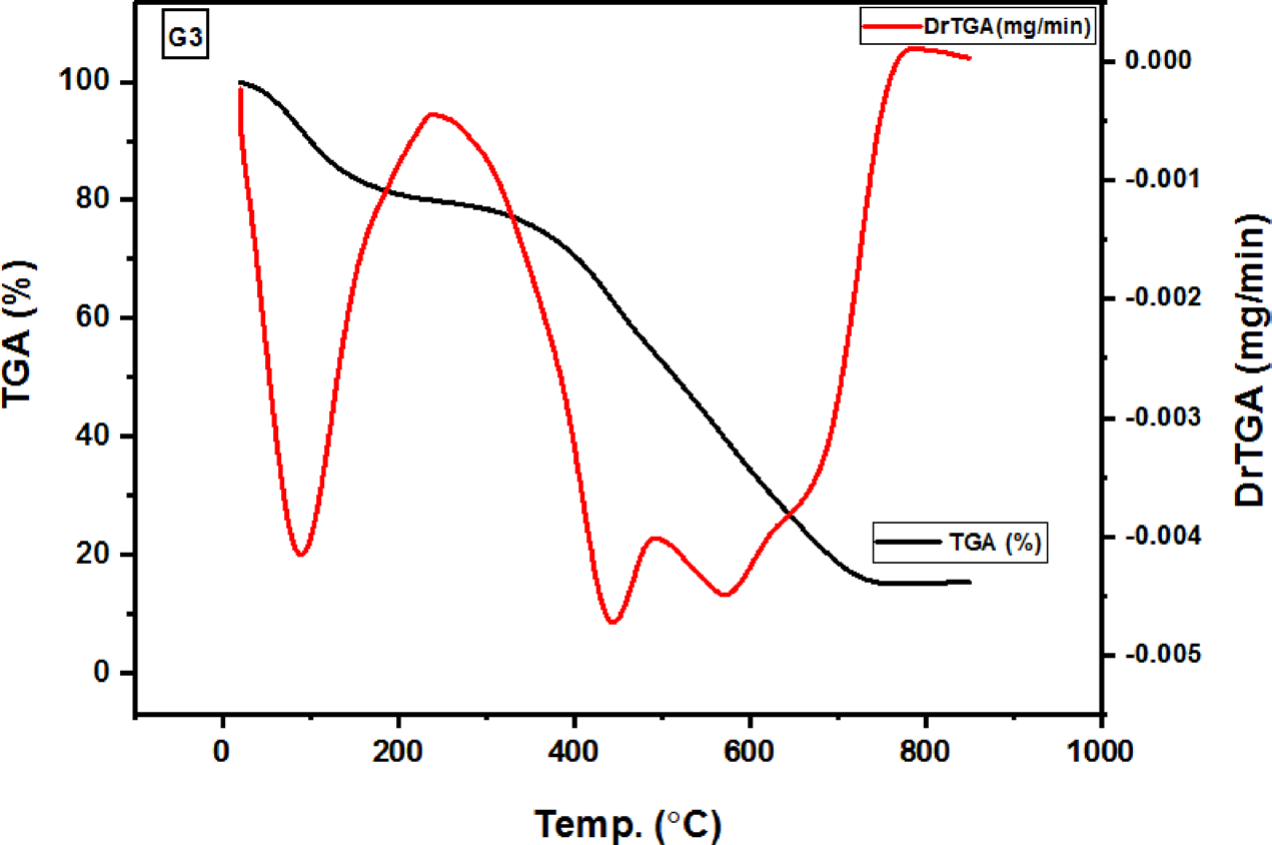

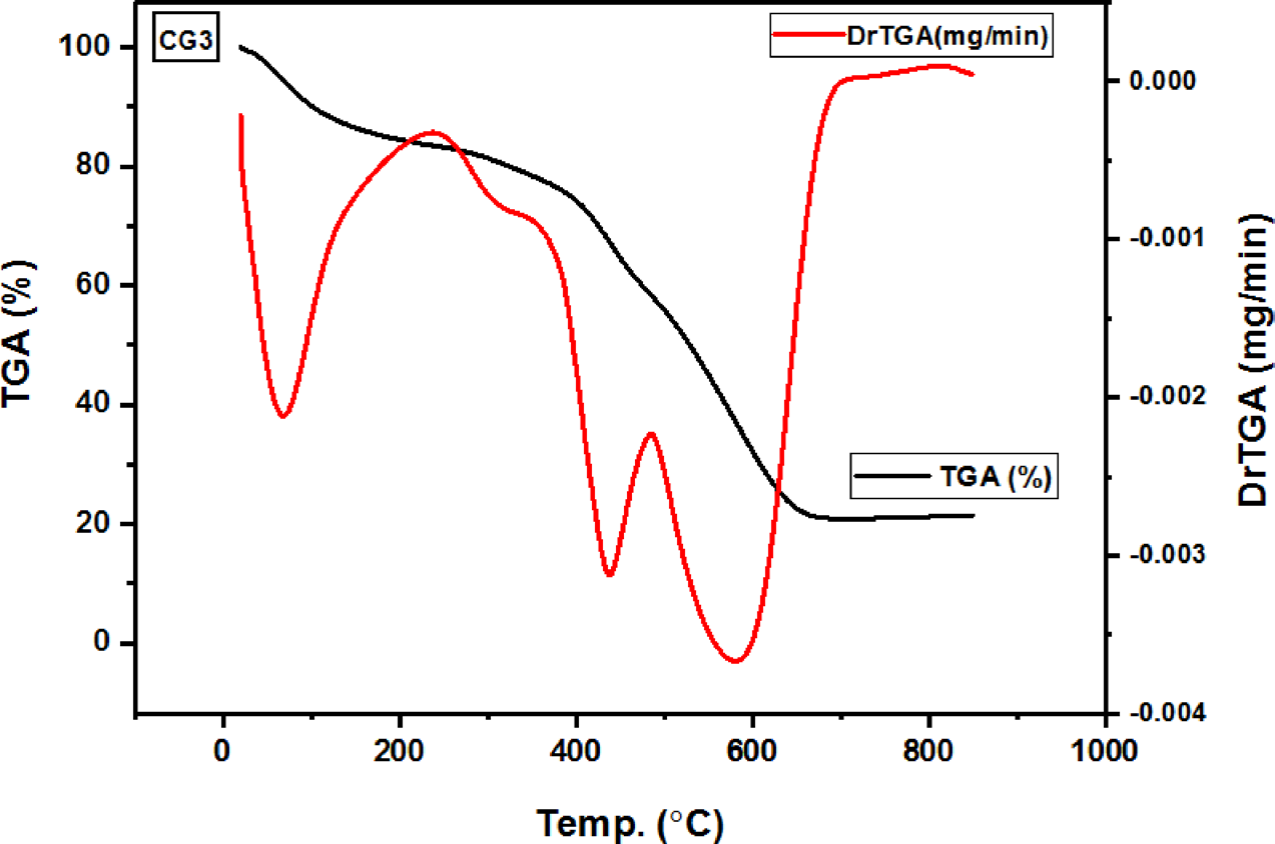
Hydrogel (G3) and the Cr(III)-hydrogel chelate (CG3) TGA thermograms.

Figure 9 shows the thermograms of hydrogel G3 (the optimal hydrogel for Cr(III) adsorption) and its corresponding CG3 (metal-hydrogel chelate).

Three stages of deterioration were revealed by the hydrogel’s (G3) TGA thermogram^60,61^. With a weight loss of 20.07%, first step is ascribed to the hydrogel’s loss of bound water at (20–240)°C^59–64^. With a weight loss of 25.53%, the second step is ascribed to the hydrogel’s degradation, which includes the deamination of Acr and NMBA and the breakdown of aromatic groups at (240–490)°C^43,65^. Third step involves the complete breakdown and chain breakdown of the polymeric hydrogel and crosslinking agents. This process speeds up after 400 °C because the hydrogel continues to absorb heat, and it finally breaks down at 755 °C, between 490 and 735 °C, with a weight loss of 38.75% because of the presence of a sulphonic group^61,65^. It was discovered that the residue was 15.65%. T_i_= 20, 240, and 490 °C, T_m_= 89.7, 444, and 571.6 °C, C_m_= 79.9, 54.4, and 15.65%, r_m_= 0.0042, 0.0047, and 0.0045 mg min^− 1^, T_f_= 735 °C with T_h_= 436 °C were the parameters that were determined.

Three stages of deterioration were visible in the Cr(III)-adsorbed hydrogel (CG3)’s TGA thermogram^61,62^. With a weight loss of 16.34%, step one is ascribed to the hydrogel’s loss of bound water at (20–230)°C^59–64^. With a weight loss of 25.03%, step two is ascribed to the complete breakdown of the polymer, which includes the deamination of Acr and NMBA and the breakdown of aromatic groups at (230–483)°C^42,65^. The hydrogel backbone of the polymeric hydrogel and crosslinking agents undergoes chain breakdown and complete degradation in step three. This process accelerates beyond 400 °C because of the metal-hydrogel chelate creation. The hydrogel continues to absorb heat and eventually degrades thermally at 700 °C, at 483–690 °C, with a reduction in weight reaching 37.74%. This is due to the presence of a sulphonic group^60,61^. It was discovered that the residue at 755 °C was 21.65%, may be attributed to the adsorption Cr(III), confirming that CG3 is more thermal stable than C3, and the formation of the complex^62^. T_i_= 20, 230, and 483 °C, T_m_= 68.44, 438.14, and 580.4 °C, C_m_= 83.66, 58.6, and 20.89%, r_m_= 0.0021, 0.0031, and 0.0037 mg min^− 1^, T_f_= 690 °C with T_h_= 376 °C were the computed parameters.

### X-ray diffraction

Figure 2S displays the G3 hydrogel and CG3 (Cr(III)-hydrogel chelate) XRD patterns. Small peaks were seen in the G3 at approximately 2θ = 25.6°, 30.9°, and 43.9°. While the G3 pattern displayed a peak at approximately at 2θ = 20.32°, which notably vanished from the CG3 XRD pattern.

The peak at 2θ = 9.16° still exist in the CG3 pattern and in the G3^60,61^. In addition, two peaks at 2θ = 53.84° and 77.56° appeared in the CG3 pattern that did not exist in the G3 pattern, corresponding to crystal planes (200), and (211). According to this, the Cr(III) adsorbed onto the prepared hydrogels (poly(Acr/Sty)) was confirmed, creating the Cr-poly(Acr/Sty) chelate.

#### G3 hydrogel swelling proportion

Equation (2) was used to estimate the hydrogel’s (G3) swelling proportion. The G3 hydrogel had an extremely high swelling proportion (10490.46%). Because hydrogel swelling is caused by electrostatic repulsion amongst the ionic charges of its interrelated species, it contains hydrophilic functional groups that can bind easily bind with water molecules^43,65^.

### Post-adsorption infrared spectral studies

**Fig. 10:**
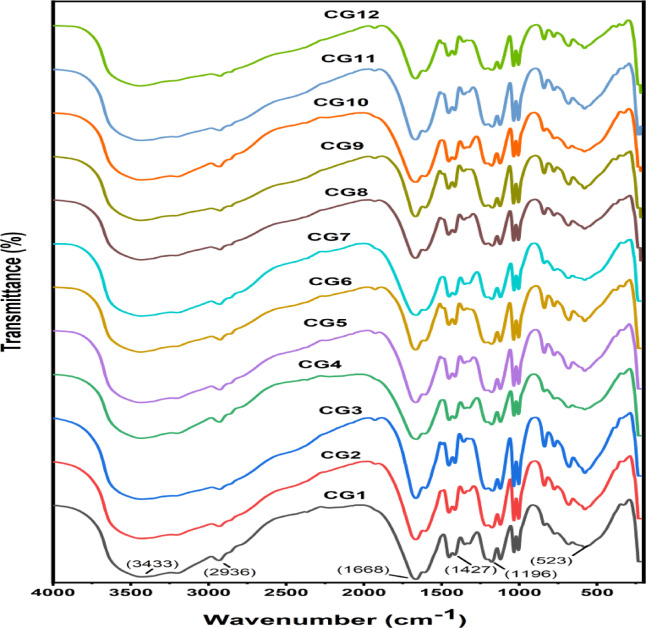
The CG1-CG12 Cr(III) adsorbed onto hydrogels FT-IR spectra.

FT-IR spectra were applied to analyze the functional groups of the metal chelates that were being studied. The CG1–CG12 (Cr-poly(Acr/Sty)) spectra are displayed in Fig. 10. Table S2 documents the assignment of the bands that are being examined. The creation of the polymeric hydrogels adsorbed with metal ions was demonstrated by the IR spectra of the C = C group disappearing, which was observed for the Acr, Sty, and NMBA attributed to symmetric and asymmetric stretching^42^ in the spectra of polymeric hydrogels adsorbed with metal ions. In the 2857–2870 cm^− 1^ range, the distinctive bands of absorption of the (CH_2_, CH) groups were identified for the methylene bending and stretching vibrational motions for the polymeric chain of the chelate^33,63^.

Regarding the Acr and NMBA spectra, the NH_2_ and NH groups showed up as peaks; the NH_2_ peaks are still present in the metal-chelates’ spectra; however, they have moved to a longer wavenumber in the 3415–3425 cm^− 1^ range, which is attributed to asymmetric stretching vibration^25,46,49^. The 1675 and 1660 cm^− 1^ peaks for the spectra of Acr and NMBA represent the carbonyl groups designated for stretching vibration. Incase of polymeric hydrogels adsorbed with metal ions, these peaks appeared but were displaced to a lower wavenumber at 1651–1659 cm^− 1^ range [44].Peaks in the Sty spectra that emerged at 690 cm^− 1^ were attributed to the stretching vibration that is asymmetric of the C-SO_3_ spectra^54^. The hydrogels’ stretching vibration spectra, which were obtained between 609 and 628 cm^− 1^, showed these characteristic peaks switching to a wavenumber that is lower. The peaks at 1132 and 1188 cm^− 1^ for the Sty spectra attributed to stretching vibration are indicative of S = O^55,56^.These peaks still present in the hydrogels’ spectra, but their wavenumber shifts to the ranges of 1184–1197 and 1114–1124 cm^− 1^. The hydrogels’ spectra showed them, but they were moved to a lower wavenumber in the region of 1602–1610 cm^− 1^, which is the distinctive absorption band of the CAr–CAr at 1630 cm^− 1^ of Sty^55^. The peaks, which were present in the hydrogels’ spectra but were moved to a longer wavenumber at 1421–1427 cm^− 1^ range, occurred at 1400 cm^− 1^ and corresponded to stretching vibration in the aromatic skeleton of styrene^55^. However, the emergence of a new band in the 518–528 cm^− 1^ area of the metal chelates’ far infrared spectra was likewise unmistakable proof that the M–O bond had formed^25,49^.

**Scheme 1:**
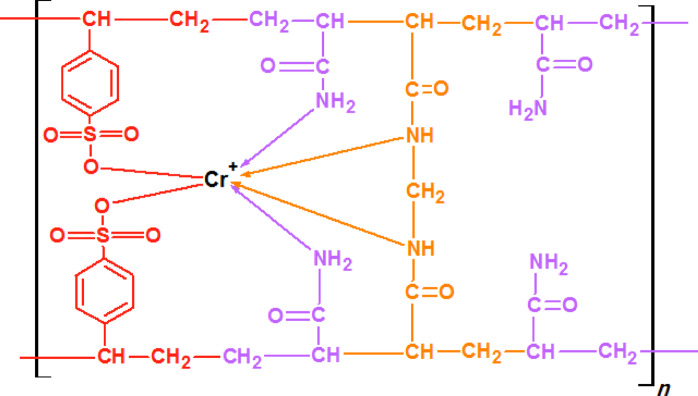
Schematic presentation for the interaction of poly(Acr/Sty) hydrogels with the Cr(III).

Scheme 1 shows a schematic illustration of the structure of the hydrogel (poly(Acr/Sty)) and its potential binding modes with Cr(III). It is believed that the interaction is caused by the existence of functional groups of the Acr and Sty monomer with carbonyl, amide, or amino groups that may interact through coordination bonds as well as the -SO_3_^−^ ionized groups of the sty monomer.

#### Adsorption kinetics

**Fig. 11:**
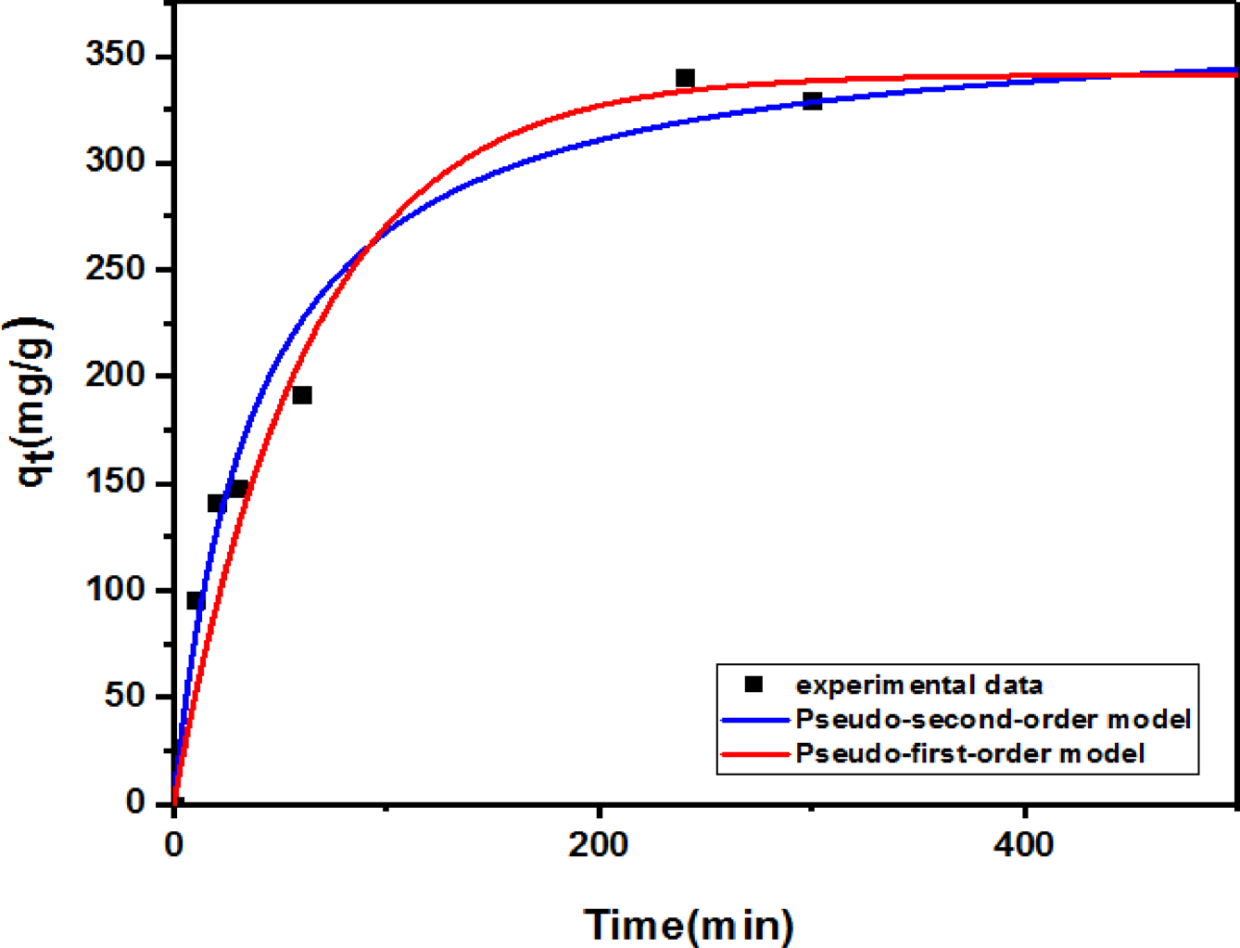
Graphs of non-linear secondorder (pseudo)and first order (pseudo)kinetics for adsorption of Cr(III) onto G3 hydrogel (poly(Acr/Sty)).

Non-linear versions of pseudo first-order and pseudo second-order kinetics were investigated. In the experiment, contact time (t) affects the amount of adsorbed Cr(III) (q_t_) on G3 hydrogel (poly(Acr/Sty)). Consequently, the q_t_ in mgg^− 1^ was plotted versus t in min, as presented in Fig. 11.

 Table 1 Represents a list of the established parameters. The correlation coefficient (R2) values and other results demonstrated that Pseudo second-order was more appropriate and more suitable for the adsorption process. The Pseudo second-order rate of kinetics was more prevalent than Pseudo first-order kinetics. This result suggested that chemisorption was obeyed by the adsorption mechanism. There was only a slight difference in the sorption capacity between the Pseudo first-order and Pseudo second-order rates when comparing the experimental values (q_e_) of Cr(III) adsorbed with their corresponding estimated q_t_ values^34,66^. Furthermore, it is clear from the results that the maximum adsorption of the Cr(III) at equilibrium in the case of the second-order is higher than in the first-order case, and so does the rate constant.

**Table 1:** Cr(III) adsorption onto G3 hydrogel kinetic parameters.

First order (pseudo)			Second order (pseudo)			
K _1_ (min ^− 1^)	q _e_ cal (mgg ^− 1^)	R ^2^	q _e_ exp (mg g^− 1^)	K _2_ (gm g ^− 1^ mi^− 1^)	q _e_ cal (mg g ^− 1^)	R ^2^
0.0157 ± 0.0016	341.38 ± 0.9006	0.9914	344.7600	7.031 ± 2.4850	370.34 ± 15.07	0.9994

#### Influence of Cr(III) initial concentration on sorption

**Fig. 12:**
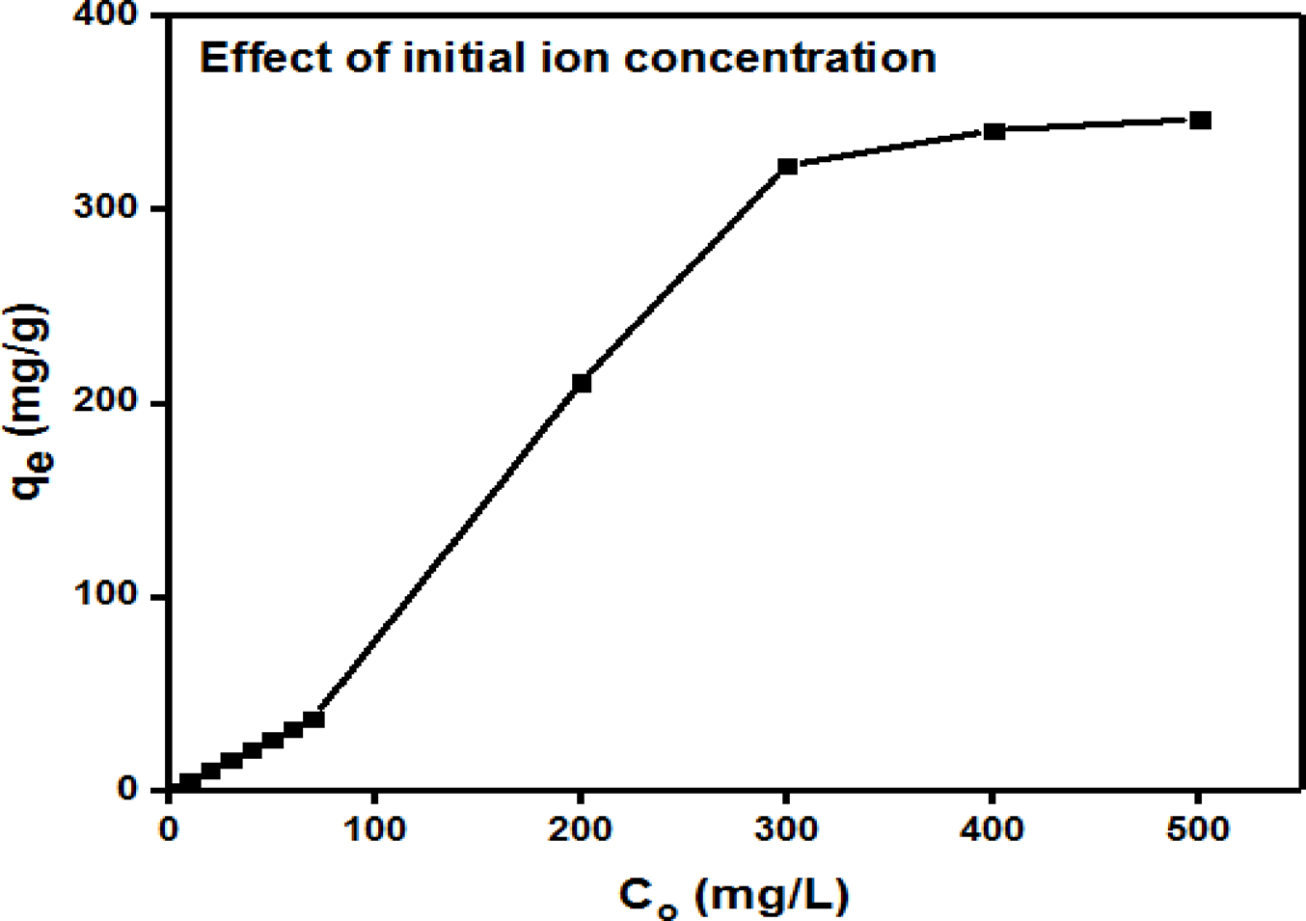
Effect of initial concentration of Cr(III) ions (10–500ppm)on adsorption onto G3 hydrogel.

Based on the results, Fig. 12 shows a graph that plots the initial Cr(III) ion concentrations, C_o_ against the amount of adsorbed Cr(III) onto G3 hydrogel, q_e_. The results demonstrated that when the original Cr(III) concentration increased, so did G3 hydrogel adsorption ability towards chromium ions using concentrations (10, 20, 30, 40, 50, to 500ppm). As the metal ion concentration increased beyond 10ppm, to less than 300 ppm the increase is almost linearly. But beyond that the hydrogel absorbed more metal until it reached adsorption saturation, or full occupation of all the available surface active sites, at 500 mg/L^25,37^. According to calculations, G3 hydrogel highest experimental adsorption capacity reached 346.48 mgg^− 1^ for dry gel.

#### Cr(III) adsorption onto G3 hydrogel isotherms

**Fig. 13:**
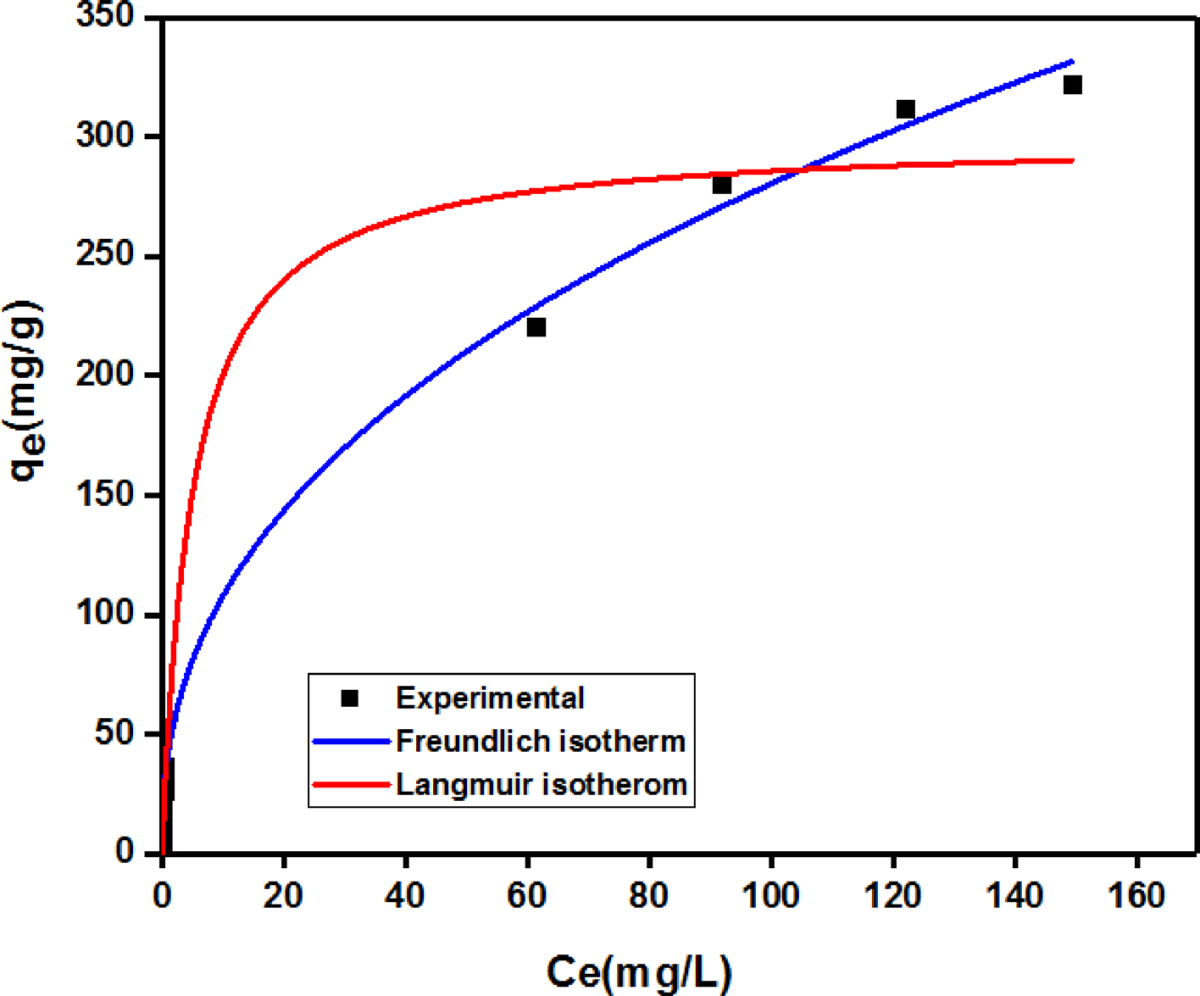
Non-linear Freundlich and Langmuir isotherms for Cr(III) ion adsorption onto G3.

Equations (5, 7) were utilized to apply the Cr(III) equilibrium adsorption data onto G3 hydrogel to non-linear Freundlich and Langmuir isotherms. The non-linear plot of q_e_ vs. C_e_ for the Freundlich and Langmuir equations is shown in Fig. 13. Table 2 contains the information gathered from the presentation of both non-linear isotherms. The data collected demonstrated that the Freundlich model had strong R^2^ values. The Langmuir isotherm did not suit the experimental data very well. Furthermore, in the experiment, the highest possible adsorption capacity of G3 hydrogel for Cr(III) was 322.47 mg/g, while the computed maximum adsorption capacity was found to be 299.9 ± 15.47 mg/g. Where the n value was larger than one suggested the advantageous adsorption with the poly(Acr/Sty) hydrogel’s heterogeneous surface, which led to the formation of multilayer, and reversible adsorption as demonstrated by the Freundlich isotherm, which also indicates that this model fits the experimental data well^37^. However, it was also discovered that the Langmuir’s model RL was less than one^40,41^.

**Table 2:** Non-linear Freundlich and Langmuir isotherms parameters for adsorption of Cr(III) on G3.

Langmuir					Freundlich		
K_L_	q_m_ cal (mgg^− 1^)	q_m_ exp (mgg^− 1^)	R^2^	R_L_	K_f_	n	R^2^
0.2 ± 0.118	299.9 ± 0.15.47	322.47	0.9725	< 1	41.2 ± 3.5	2.4 ± 0.105	0.9974

### Cr(III) desorption from G3 hydrogel

**Fig. 14:**
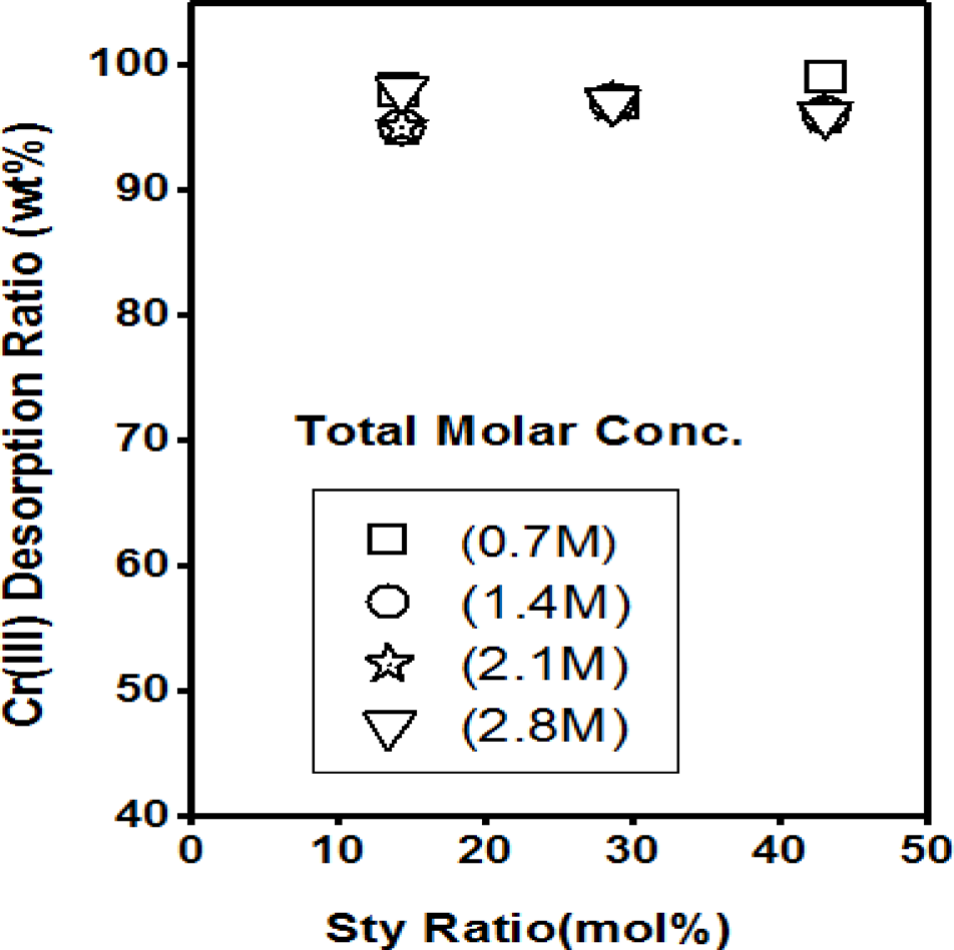
Cr(III)desorption from different hydrogels (poly(Acr/Sty) of total molar concentrations 0.7 M, 1.4 M, 2.1 M, and 2.8 M.

The Sty-dependence of the Cr(III) desorbed from the poly(Acr/Sty) hydrogels with varying network concentrations was displayed in Fig. 14. It displayed the outcomes of the desorption operations, where the used hydrogels were (CG1 - CG12) of total molar concentrations (0.7 M − 2.8 M). The desorbed Cr(III) was almost 100%, according to the results. The most significant discovery is that the hydrogels returned to their initial form after at least four attempts, demonstrating their resilience and possibly allowing for further use in capturing additional Cr(III) ions. This is consistent with the results obtained from the kinetic adsorption and the adsorption isotherm studies that indicated that the adsorption is chemisorption and reversible.

### Adsorption study of the Cr(III) industrial tannery wastewater onto (AAm/Sty)hydrogels

**Fig. 15:**
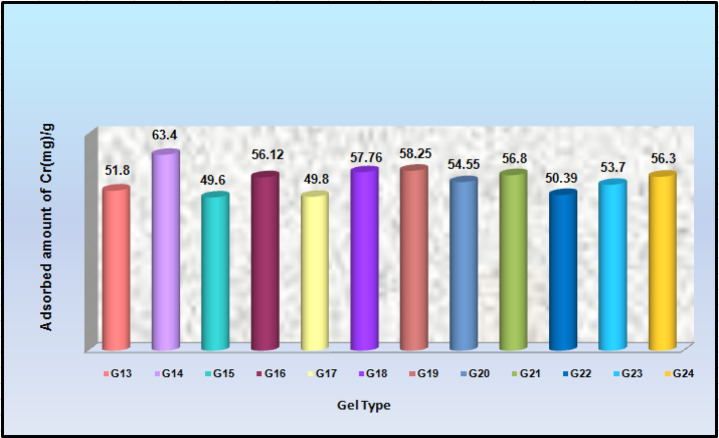
Amount of adsorbed Cr(III) from industrial tanning wastewater onto poly(Acr/Sty) hydrogels.

A comparison graph was conducted to compare the adsorbed amount of Cr(III) from industrial tanning wastewater onto the prepared poly(Acr/Sty) hydrogels in shown in Fig. 15. It is obvious that the highest quantity of adsorbed Cr(III) capture was found to be the hydrogel at a total molar concentration of 0.7 M. and reached 63.4 mgg-1 of dry hydrogel. It is attributed to the presence of other metal ions and contaminants in the tanning wastewater.

## A comparison between the present study and relevant literature

For the assessment of poly(Acr/Sty) hydrogel’s performance, comparing it to other widely used adsorbents is crucial for assessing environmental impact, cost-effectiveness, adsorption capacity, regeneration ability, and selectivity, among other crucial factors. Table 3 compares the adsorption capacity of poly(Acr/Sty) hydrogel with that of comparable adsorbents. The benefits and drawbacks of poly(Acr/Sty) hydrogel have been highlighted using other popular adsorbents in relation to the mentioned important characteristics shown in (Table 4). Because of their high adsorption capacity, the poly(Acr/Sty) hydrogels may be effectively employed to remove Cr(III) ions, which are present in tanning wastewater effluents, from their aqueous solution. The poly(Acr/Sty) hydrogels may have an impact on reducing pollution.

**Table 3:** Poly(Acr/Sty) hydrogel performance compared to previous known adsorbents.

Adsorbent	Pollutant	Capacity of adsorption in mg g^− 1^	Refs.	Notes
Hydrogels of poly(acrylamide/sodium acrylate)	Chromium (heavy metal)	206.3	^2^	Moderate cost and high affinity for metals
Nanofiber of poly(acrylonitrile co maleic acid)	Cr(VI) and Ni(II) (heavy metals)	174.0 and 218.9, (25 min contact time)	^57→ 58^	Moderate cost and high affinity for metals
Zeolites	Ammonium	100 − 20	^59→ 67^	Excellent ions exchange, but reduced capacity
Composites of Bentonite-Clay	Metals and dyes	150 − 30	^60,61→ 68,61,69^	Low cost, but with lower affinity for metals
Adsorbents based on Chitosan	Dyes and metals	200 − 80	^62 → 70^	Less stable and biodegradable
PUL-g-P(AA-co-AM)hydrogel	Cu(II), Ni(II), and Cd(II) (heavy metals)	154.00, 126.75, and 146.90	^63→ 71^	Moderate cost and high affinity for metals
Activated Carbon	Organic pollutants, metals	300 − 50	^64,65→72 73^	Highly porous but less selective
Poly(Acr/Sty) hydrogel	Chromium (heavy metal)	346.48	Present work	Great affinity, selective and effective for metals with very low cost

**Table 4:** The limitations and advantages of poly(Acr/Sty) hydrogels vs. diverse adsorbents.

Criteria	Selectivity	Cost & Availability	Regeneration & Reusability	Refs.	Environmental Impact & Stability
Poly(Acr/Sty) hydrogel, ionic hydrogels (Present study)	Great selectivity for particular metallic ions	Low cost due to reusability	Reusable at least four times, retains 98% capacity, acid/base regeneration	–	Stable, non-toxic, sustainable
Hydrogels of poly(acrylamide/sodium acrylate)	Great selectivity for particular metallic ions	Low cost due to reusability	Reusable at least four times, retains 98% capacity, acid/base regeneration	^2^	Stable, non-toxic, sustainable
Bentonite	Low to moderate	Very low	Moderate	^67,68,68,69^	Benign
Chitosan	Great selectivity for particular metallic ions	High cost due to processing	Degrades over multiple cycles	^69→ 70^	Biodegradable. low toxicity
Zeolites	Good, due to ion exchange and uniform pore size	Moderate cost	Good chemical stability and moderate regeneration,	^70→ 67^	Generally safe
Activated Carbon	Absorbs pollutants in a wide range	Moderate to high, especially for high-grade forms	Energy intensive by possible thermal regeneration	^71→ 73,74 74]^	Safe but high carbon footprint

## Conclusions

To remove Cr(III), poly(Acr/Sty) hydrogel adsorbents were successfully created and used in this study. The resulting hydrogel series showed that the adsorption capacity is influenced by the monomer’s molar ratio. The poly(Acr/Sty) hydrogel’s remarkable shape change and maximum adsorption capacity (346.48 mg g^− 1^) were established by the adsorption method. Significantly, the hydrogel’s ability to capture chromium ions is significantly impacted by the amount of Sty monomer present. Modern analytical methods such as X-ray diffraction (XRD), energy dispersive X-ray (EDX), scanning electron microscopy (SEM), Furrier transform infrared spectroscopy (FT-IR), and thermal gravimetric analysis (TGA) were used to analyze Cr-poly(Acr/Sty) and poly(Acr/Sty) hydrogels. Hydrogels displayed the maximum adsorption capacity for Cr(III) ions, with a dry gel adsorption capacity of 346.48 mg g^− 1^. Their adsorption performance is in line with the Freundlich isotherm, and their adsorption kinetics are consistent with the pseudo second-order approach. The excellent recovery percentage (almost 100 weight%) indicates their potential for reuse in environmental applications, such as wastewater treatment. The produced hydrogels with effective capturing capability were subjected to adsorption utilizing Cr(III) ions from tanning industrial wastewater with high efficiency. The poly(Acr/Sty) hydrogel could prevent endangering human health and the environment from the harm of chromium and may have a wide range of uses for environmental purification, which could lead to cleaner water sources and more environmentally friendly, sustainable industrial processes.

## Supplementary Information

Below is the link to the electronic supplementary material.

Supplementary Material 1

## Data Availability

This published article [together with its supplemental information files] contains all data created or examined during this investigation.
