# Supplementary material for: Poly(acrylamide/styrenesulfonic acid) hydrogels for effective removal of Cr(III) ions
Source: Sci Rep. 2025 Dec 18;15:44133. doi: 10.1038/s41598-025-30812-w (PMC12717189; doi:10.1038/s41598-025-30812-w)
Supplement: Supplementary file 1 — Supplementary Material 1 [file 41598_2025_30812_MOESM1_ESM.docx]

**Application of poly(acrylamide/styrenesulfonic acid) hydrogel as highly effective adsorbent in recovery and reuse of chromium**

**Abdalla M. Khedr, Ashraf A. Lotfalla(✉), Kazuhiro Hara, Satoru Yoshioka**and **Mohamed Gaber**

**Supplementary materials**

**Table S1 shows the amount of Cr(III) adsorbed after each time interval for each prepared poly(AAm/Sty) hydrogel**

**and the molar ratios of each monomer with 0.133wt% crosslinker**

|  | **Total Molar Conc.** | **AAm%**  **(mol%)**  **Sty%**  **(mol%)** | | **Molar Ratio**  **(AAm:SA)M** | **Gel** | **Time(hours)** | | | | | | | | |
| --- | --- | --- | --- | --- | --- | --- | --- | --- | --- | --- | --- | --- | --- | --- |
|  |  |  |  |  |  | **0.17** | **0.33** | **0.5** | **1** | **2** | **3** | **4** | **5** | **24** |
| **AdsorbedCr(III) (mg/g)** | **0.7M** | 86 | 14 | 0.6:0.1 | **G1** | 2.4 | 1.70 | 1.69 | 2.10 | 2.45 | 2.33 | 2.52 | 2.48 | 2.35 |
|  |  | 71 | 29 | 0.5:0.2 | **G2** | 1.79 | 2.21 | 2.54 | 2.16 | 2.25 | 2.43 | 2.42 | 2.30 | 2.88 |
|  |  | 57 | 43 | 0.4:0.3 | **G3** | 0.90 | 133 | 1.40 | 1.81 | 3.14 | 3.27 | 3.21 | 3.11 | 3.27 |
|  | **1.4M** | 85.7 | 14.3 | 1.2:0.2 | **G4** | 1.83 | 1.90 | 1.85 | 1.43 | 2.28 | 1.98 | 2.54 | 2.46 | 2.68 |
|  |  | 71.7 | 28.6 | 1.0:0.4 | **G5** | 1.85 | 1.96 | 2.03 | 1.59 | 2.06 | 2.38 | 2.14 | 2.42 | 2.69 |
|  |  | 57 | 43 | 0.8:0.6 | **G6** | 1.65 | 1.81 | 1.56 | 1.80 | 1.82 | 2.40 | 2.53 | 2.32 | 2.70 |
|  | **2.1M** | 85.7 | 14.3 | 1.8:0.3 | **G7** | 1.79 | 1.60 | 2.06 | 1.88 | 1.86 | 2.43 | 2.53 | 2.30 | 2.18 |
|  |  | 71.4 | 28.6 | 1.5:0.6 | **G8** | 1.33 | 1.89 | 1.73 | 1.45 | 2.13 | 2.03 | 2.39 | 2.19 | 2.25 |
|  |  | 57 | 43 | 1.2:0.9 | **G9** | 1.43 | 1.73 | 1.75 | 1.93 | 1.66 | 2.15 | 2.21 | 2.41 | 2.62 |
|  | **2.8M** | 85.7 | 14.3 | 2.4:0.4 | **G10** | 1.49 | 1.41 | 1.81 | 1.85 | 1.74 | 2.38 | 2.19 | 2.40 | 2.50 |
|  |  | 71.4 | 28.6 | 2.0:0.8 | **G11** | 1.60 | 1.67 | 1.73 | 1.99 | 2.13 | 2.24 | 2.42 | 2.51 | 2.59 |
|  |  | 57 | 43 | 1.6:1.2 | **G12** | 1.66 | 1.68 | 1.66 | 1.74 | 2.00 | 2.14 | 2.30 | 2.39 | 2.83 |

**Table S2 The important assigned bands in the FTIR spectra of the monomers used (AAm, Sty, and MBA), poly(AAm/Sty) hydrogels (G1–G12) and Cr-poly(AAm/Sty) metal complex (CG1–CG12)**

| **Compound** | **Wavenumber cm^-1^** | | | | | | | | | |
| --- | --- | --- | --- | --- | --- | --- | --- | --- | --- | --- |
|  | **ʋ _NH2_** | **ʋ _NH_** | **ʋ _C = C_** | **ʋ _C = O_** | **ʋ _CH2, CH_** | **ʋ _C-SO3_** | **ʋ _S = O_** | **ʋ _(C -C)Ar_** | **ʋ _Aromatic skeletal_** | **ʋ _M → O_** |
| **AAm** | 3351 | --- | 3107 | 1675 | --- | --- | --- | --- | --- | --- |
| **Sty** | --- | --- | 3087 | --- | --- | 690 | 1188,1132 | 1630 | 1400 | --- |
| **MBA** | --- | 3308 | 3101 | 1660 | 2956 | --- | --- | --- | --- | --- |
| **G1** | 3440 | 3203 | --- | 1668 | 2939,2862 | 682 | 1197,1120 | 1602 | 1413 | --- |
| **CG1** | 3433 | 3190 | --- | 1668 | 2935,2796 | 622 | 1195,1122 | 1608 | 1427 | 522 |
| **G2** | 3440 | 3192 | --- | 1668 | 2927,2856 | 682 | 1195,1120 | 1606 | 1412 | --- |
| **CG2** | 3421 | 3195 | --- | 1670 | 3933,2796 | 619 | 1191,1118 | 1606 | 1421 | 526 |
| **G3** | 3438 | 3200 | --- | 1668 | 2937,2860 | 688 | 1195,1126 | 1606 | 1413 | --- |
| **CG3** | 3427 | 3197 | --- | 1668 | 2947,2794 | 611 | 1186,1120 | 1610 | 1425 | 524 |
| **G4** | 3440 | 3194 | --- | 1668 | 2931,2854 | 688 | 1193,1126 | 1602 | 1409 | --- |
| **CG4** | 3433 | 3197 | --- | 1666 | 2941,2790 | 628 | 1184,1122 | 1604 | 1425 | 518 |
| **G5** | 3446 | 3200 | --- | 1668 | 2931,2866 | 684 | 1195,1124 | 1608 | 1411 | --- |
| **CG5** | 3437 | 3195 | --- | 1670 | 2930,2781 | 617 | 1184,1118 | 1604 | 1423 | 528 |
| **G6** | 3438 | 3213 | --- | 1668 | 2929,2858 | 682 | 1195,1122 | 1602 | 1414 | --- |
| **CG6** | 3429 | 3197 | --- | 1668 | 2937,2783 | 611 | 1195,1114 | 1602 | 1421 | 522 |
| **G7** | 3435 | 3201 | --- | 1670 | 2933,2860 | 682 | 1193,1128 | 1608 | 1409 | --- |
| **CG7** | 3435 | 3195 | --- | 1670 | 2931,2796 | 621 | 1193,1120 | 1606 | 1423 | 524 |
| **G8** | 3442 | 3203 | --- | 1668 | 2929,2858 | 688 | 1195,1122 | 1602 | 1413 | --- |
| **CG8** | 3431 | 3190 | --- | 1668 | 2931,2798 | 617 | 1197,1116 | 1608 | 1421 | 528 |
| **G9** | 3448 | 3201 | --- | 1670 | 2925,2862 | 684 | 1197,1120 | 1604 | 1413 | --- |
| **CG9** | 3427 | 3190 | --- | 1668 | 2935,2788 | 615 | 1195,1116 | 1608 | 1423 | 526 |
| **G10** | 3429 | 3194 | --- | 1670 | 2933,2862 | 688 | 1190,1118 | 1612 | 1413 | --- |
| **CG10** | 3440 | 3190 | --- | 1668 | 2937,2794 | 624 | 1190,1116 | 1606 | 1423 | 520 |
| **G11** | 3431 | 3195 | --- | 1670 | 2937,2860 | 682 | 1199,1128 | 1606 | 1409 | --- |
| **CG11** | 3427 | 3195 | --- | 1669 | 2935,2780 | 615 | 1193,1120 | 1610 | 1425 | 524 |
| **G12** | 3440 | 3200 | --- | 1670 | 2931,2864 | 686 | 1199,1124 | 1604 | 1411 | --- |
| **CG12** | 3437 | 3190 | --- | 1668 | 2935,2790 | 609 | 1195,1124 | 1608 | 1423 | 526 |


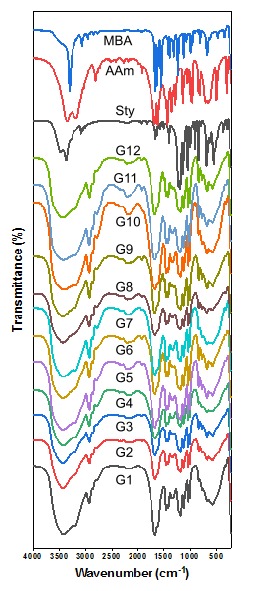
**Fig. S1.** FTIR spectra for monomers (AAm, MBA, Sty) and synthesized hydrogels (G1-G12)


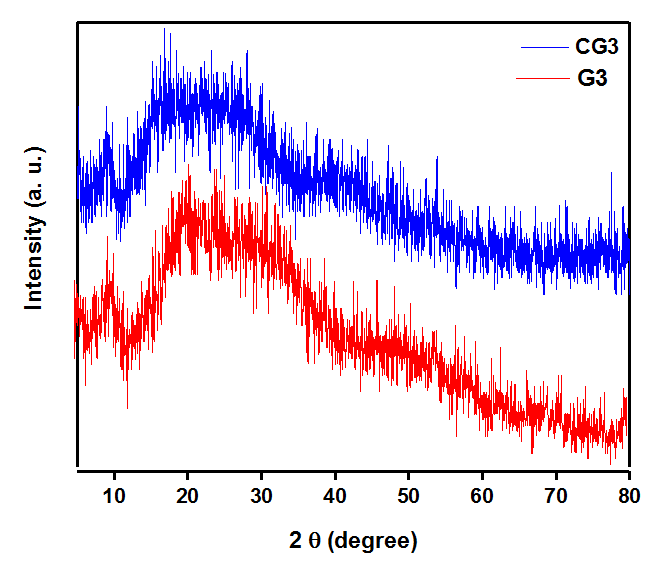


**Fig. S2.** XRD diffraction pattern of hydrogel (G3) and Cr(III)-hydrogel complex (CG3)
